# Nociplastic Pain: A Critical Paradigm for Multidisciplinary Recognition and Management

**DOI:** 10.3390/jcm13195741

**Published:** 2024-09-26

**Authors:** Jacob N. Ablin

**Affiliations:** Tel Aviv Sourasky Medical Center, Tel Aviv University, 6 Weizmann St., Tel Aviv 69978, Israel; jacobab@tlvmc.gov.il

**Keywords:** nociplastic pain, fibromyalgia, chronic pain

## Abstract

Our understanding of chronic pain has evolved significantly, shifting from a focus on peripheral damage to recognizing the central mechanisms underlying pain perception. This perspective article explores the concept of nociplastic pain, a term introduced by the International Association for the Study of Pain (IASP) in 2017, which describes pain arising from altered pain modulation within the central nervous system, without clear evidence of tissue damage or inflammation. The historical progression from fibrositis to fibromyalgia, and now to nociplastic pain, underscores the complexity of chronic pain syndromes and the need for a multidisciplinary approach to management. Nociplastic pain is characterized by central sensitization, leading to heightened pain sensitivity and often accompanied by comorbidities such as fatigue, sleep disturbances, and cognitive difficulties. Advances in neuroimaging have revealed altered connectivity within key brain networks, such as the default mode and salience networks, in patients with nociplastic pain, providing insights into the neural underpinnings of this condition. The article also addresses controversies surrounding the role of small fiber neuropathy and autonomic dysfunction in nociplastic pain, highlighting the ongoing debates in the field. The practical importance of recognizing nociplastic pain across various medical disciplines—including primary care, orthopedics, neurology, psychiatry, and rheumatology—is emphasized, with recommendations for integrating this knowledge into clinical practice. Emerging therapies, such as neurofeedback, hyperbaric oxygen therapy, and neuromodulation, offer new avenues for treatment, particularly for patients who do not respond to conventional approaches. The article calls for continued research into the mechanisms of nociplastic pain, the development of reliable diagnostic tools, and the exploration of novel therapeutic strategies to improve patient outcomes. The recognition and management of nociplastic pain are crucial for advancing the care of patients with chronic pain, necessitating interdisciplinary collaboration and a patient-centered approach.

## 1. Introduction

The understanding of chronic pain has undergone a profound transformation over the past century, evolving from a simplistic view focused solely on peripheral damage to a more nuanced recognition of complex central mechanisms [1]. One of the most important breakthroughs in understanding chronic pain mechanisms came from the field of psychoneuroimmunology, particularly the work of Watkins and Maier, who explored the links between central sensitization and sickness behavior [2,3]. Their research demonstrated how central nervous system (CNS) alterations can drive pain and behavioral responses in the absence of ongoing peripheral pathology, laying the groundwork for the later development of concepts like central sensitization and nociplastic pain [4]. Central sensitization, which refers to the heightened responsiveness of the CNS to sensory stimuli, has been identified as a key mechanism not only in fibromyalgia [5], but also in other chronic pain conditions, such as osteoarthritis [6], low back pain [7], post-cancer pain [8], and even postoperative pain syndromes [9,10]. These conditions, although traditionally viewed through the lens of nociceptive or neuropathic mechanisms, are now increasingly recognized as involving nociplastic components. The introduction of the nociplastic pain concept by the International Association for the Study of Pain (IASP) in 2017 marked a significant leap forward [11,12]. This new pain category emphasizes pain arising from altered pain modulation within the CNS, without clear evidence of tissue damage or inflammation. Importantly, the nociplastic pain framework broadens our understanding of chronic pain, acknowledging that conditions like osteoarthritis, low back pain, and neck pain can also involve central sensitization, thereby shifting the focus beyond fibromyalgia alone. The clinical identification of the central sensitization pain phenotype has been a critical development in the journey toward recognizing nociplastic pain. Many influential research efforts have aimed to define this phenotype, linking it with widespread or localized pain, fatigue, and cognitive symptoms, thus contributing to the creation of the nociplastic pain definition. The big breakthrough in the field has been the recognition that nociplastic pain is highly prevalent in both widespread pain conditions, like fibromyalgia, and non-widespread conditions, like osteoarthritis, lower back pain, and post-cancer pain [13].

This article seeks to explore the implications of recognizing nociplastic pain across a wide spectrum of chronic pain conditions, emphasizing its prevalence and impact on clinical practice in various medical disciplines. Understanding nociplastic pain is essential not only for conditions like fibromyalgia, but also for the effective management of other chronic pain disorders that involve central sensitization.

As we explore the mechanisms, clinical presentations, and treatment approaches for nociplastic pain, this perspective aims to highlight its critical importance across multiple medical specialties. By fostering a multidisciplinary understanding of nociplastic pain, we can improve patient outcomes and address a significant unmet need in chronic pain management.

## 2. Understanding Nociplastic Pain

Nociplastic pain represents a fundamental shift in our understanding of chronic pain mechanisms, distinguishing itself from the more traditional categories of nociceptive and neuropathic pain. This type of pain is characterized by altered pain processing in the central nervous system, leading to pain perception without clear peripheral damage or significant inflammation. Unlike nociceptive pain, which results from direct tissue injury or inflammation, and neuropathic pain, which arises from nerve damage, nociplastic pain is rooted in central sensitization—a heightened responsiveness of the central nervous system to sensory input [14].

## 3. Central Sensitization: Pathophysiology and Imaging Findings

### 3.1. Early Studies on Pain Response

Central sensitization, a concept foundational to nociplastic pain, refers to the heightened responsiveness of the central nervous system to sensory input, resulting in amplified pain perception. The origins of central sensitization in pain research can be traced to the seminal work of Clifford Woolf, who, in the 1980s, first described the phenomenon in basic science studies of spinal cord neuroplasticity and its role in chronic pain [15]. Woolf’s pioneering work demonstrated that central sensitization could be induced by persistent peripheral noxious stimuli, leading to the hyperexcitability of neurons in the dorsal horn of the spinal cord, a key mechanism in the persistence of chronic pain [16]. In addition to the research into central sensitization, the work of David Butler and Lorimer Moseley has been highly influential in advancing our understanding of pain neurobiology and the importance of pain education. Their contributions, particularly through the development of concepts like “Explain Pain”, have emphasized the critical role of educating patients about the neurobiological processes underlying chronic pain, which is essential for managing conditions like nociplastic pain [17,18].

Building on Woolf’s foundational work, clinical research into conditions like fibromyalgia provided further insights into the role of central sensitization in chronic pain syndromes. The pioneering studies by Staud and Vierck, for example, were instrumental in demonstrating central sensitization in fibromyalgia patients [19]. Their research highlighted the enhanced sensitivity to pain and exaggerated responses to stimuli that would not normally provoke pain, providing important clinical evidence for central sensitization as a key feature of fibromyalgia.

Gracely et al.’s 2002 study further advanced the field by using functional magnetic resonance imaging (fMRI) to show that fibromyalgia patients exhibited heightened brain activity in pain-processing regions, such as the insula and anterior cingulate cortex, in response to stimuli that were perceived as non-painful by healthy controls [20]. While this study was groundbreaking in providing neuroimaging evidence of abnormal central pain processing, it was part of a broader body of research that had already established central sensitization as a fundamental mechanism underlying conditions like fibromyalgia.

By incorporating both basic science discoveries and clinical research, our understanding of central sensitization has evolved to encompass not only fibromyalgia, but also a wide range of nociplastic pain conditions such as osteoarthritis, low back pain, and post-cancer pain.

### 3.2. Changes in Brain Connectivity: Resting-State and Induced

#### 3.2.1. Resting-State Connectivity

Advances in neuroimaging have revealed that patients with nociplastic pain, particularly fibromyalgia, exhibit altered resting-state connectivity in the brain [21,22,23]. Studies using resting-state fMRI have identified abnormal functional connectivity within the brain’s pain-processing networks, even in the absence of external stimuli. Key networks involved include the default mode network (DMN) [24], the salience network [25,26], and the sensorimotor networks. For example, increased connectivity within the DMN, particularly in regions like the posterior cingulate cortex and medial prefrontal cortex, has been associated with the widespread pain and cognitive dysfunction seen in fibromyalgia [27].

#### 3.2.2. Induced Connectivity

When exposed to pain stimuli, patients with nociplastic pain demonstrate exaggerated responses in these networks, indicating an overactive pain-processing system. The insula, a region critical for integrating sensory input and emotional responses, shows hyperactivity in nociplastic pain states. This hyperactivity is often coupled with increased connectivity between the insula and other regions, such as the anterior cingulate cortex and amygdala, which are involved in the emotional and affective dimensions of pain [28,29].

### 3.3. Role of Key Brain Regions and Networks

#### 3.3.1. Default Mode Network (DMN)

The DMN, typically active during rest and involved in self-referential thought processes, has been implicated in the pathophysiology of nociplastic pain. Abnormal connectivity within the DMN, particularly between the medial prefrontal cortex and the posterior cingulate cortex, correlates with the chronic pain and fatigue experienced by patients. These regions are thought to contribute to the persistent, self-focused pain experiences characteristic of nociplastic pain [30].

#### 3.3.2. Insula and Salience Network

The insula plays a central role in nociplastic pain by integrating sensory information with emotional and cognitive processes. It is part of the salience network, which helps determine the significance of stimuli and modulates attention toward them. In nociplastic pain, the insula and salience network are often hyperactive, leading to an exaggerated response to both painful and non-painful stimuli. This overactivity may explain the heightened sensitivity to various sensory inputs (e.g., light, sound) observed in these patients [29,31].

#### 3.3.3. Amygdala

The amygdala, traditionally associated with fear and emotional responses, also plays a role in nociplastic pain. Hyperconnectivity between the amygdala and regions involved in pain processing, such as the insula and anterior cingulate cortex, suggests that the emotional and affective dimensions of pain are amplified in nociplastic pain states. This may contribute to the strong emotional distress and anxiety often seen in patients with chronic pain conditions like fibromyalgia [32]. This understanding has focused attention on the amygdala as a potential therapeutic target for neuromodulation (through neurofeedback) for treating symptoms of fibromyalgia [33].

## 4. Controversies and Emerging Topics: Small Fiber Neuropathy and Autonomic Dysfunction

### 4.1. Small Fiber Neuropathy (SFN)

#### 4.1.1. The Debate

Small fiber neuropathy (SFN) is a condition characterized by damage to the small nerve fibers that are responsible for pain and temperature sensation. Some researchers have proposed that SFN may be present in a subset of patients with nociplastic pain, particularly fibromyalgia, and could partially explain the widespread pain these patients experience. Studies have found reduced intraepidermal nerve fiber density in some fibromyalgia patients, suggesting a possible neuropathic component [34].

#### 4.1.2. Controversy

The role of small fiber neuropathy (SFN) in nociplastic pain remains controversial [35]. Critics argue that while SFN may be present in some patients, it does not fully account for the central mechanisms of pain seen in nociplastic disorders. Moreover, the findings of reduced nerve fiber density are not consistent across all studies, and the clinical significance of these findings is still under debate. Some researchers believe that the observed nerve fiber changes might reflect neuroplasticity or secondary changes due to chronic pain, rather than being a primary cause of nociplastic pain [36]. Clinically, it can be challenging to “differentiate” small fiber neuropathy form fibromyalgia [37], and at any rate, unless a clear (and treatable) cause for small fiber neuropathy can be identified, the therapeutic implications are not clear. Nonetheless, the controversy around this topic is likely to carry on, as the concept of nociplastic pain evolves.

### 4.2. Autonomic Dysfunction

#### 4.2.1. The Role in Nociplastic Pain

Autonomic dysfunction, which involves the dysregulation of the autonomic nervous system, has been implicated in nociplastic pain conditions including fibromyalgia [38]. Symptoms such as orthostatic intolerance, gastrointestinal disturbances, and excessive sweating are commonly reported by patients with fibromyalgia and other nociplastic pain conditions [39]. This has led to the hypothesis that autonomic dysregulation might contribute to or exacerbate the pain experience in these patients [40].

#### 4.2.2. Current Understanding and Controversy

The link between autonomic dysfunction and nociplastic pain is still under investigation. Some studies suggest that dysregulated autonomic responses, such as abnormal heart rate variability or blood pressure changes, could contribute to the heightened pain sensitivity and fatigue seen in these patients [41,42]. However, the causal relationship between autonomic dysfunction and nociplastic pain is not yet fully understood, and more research is needed to clarify this connection. There is also debate over whether autonomic symptoms are a direct result of central sensitization or if they represent a separate, overlapping pathology.

## 5. Practical Importance of Recognizing Nociplastic Pain across Medical Disciplines

The early identification of nociplastic pain is essential for preventing unnecessary tests and treatments, and for providing timely and appropriate management. Recent advancements in clinical recommendations have provided clear criteria for identifying nociplastic pain, which healthcare professionals should be aware of. The International Association for the Study of Pain (IASP) formally endorsed clinical criteria for nociplastic pain in 2022, developed by Kosek et al., providing a framework for identifying nociplastic pain across various conditions [11]. These criteria emphasize the presence of pain without a clear nociceptive or neuropathic etiology, as well as the frequent association with comorbidities such as sleep disturbances, fatigue, and cognitive difficulties.

Additionally, several international expert groups have developed specific guidelines for nociplastic pain phenotyping in certain populations. For example, the CANPPHE recommendations for cancer survivors have highlighted the importance of distinguishing nociplastic pain from other types of pain in post-cancer pain syndromes, ensuring that these patients receive appropriate non-opioid treatments and avoid over-testing [43]. Similarly, the BACPAP recommendations for low back pain phenotyping have underscored the need for the early recognition of nociplastic pain in patients with chronic low back pain, where central sensitization plays a significant role [44]. These guidelines support the importance of holistic, multidisciplinary management strategies that prioritize non-pharmacological treatments such as exercise, cognitive-behavioral therapy (CBT), and pain education.

### 5.1. Primary Care

#### 5.1.1. Early Identification and Holistic Management

Primary care physicians are often the first point of contact for patients with chronic pain. Recognizing nociplastic pain early in the clinical encounter is vital for preventing the cascade of unnecessary tests, treatments, and referrals that often accompany chronic pain syndromes. By understanding nociplastic pain, primary care providers can take a more holistic approach to management, focusing on both the physical and psychological aspects of pain. This includes integrating non-pharmacological strategies such as exercise programs, cognitive-behavioral therapy (CBT), and sleep hygiene into the treatment plan.

#### 5.1.2. Referral and Coordination of Care

Recognizing nociplastic pain also enables primary care physicians to make more targeted referrals to specialists, such as rheumatologists, neurologists, or pain management experts, when necessary. The effective coordination of care among these providers is crucial for managing the multifaceted nature of nociplastic pain.

### 5.2. Orthopedics

#### 5.2.1. Surgical Decision-Making

In orthopedic practice, the distinction between nociceptive and nociplastic pain is particularly important when considering surgical interventions. For example, in patients with osteoarthritis, recognizing a nociplastic component can influence the decision to proceed with joint replacement surgery [45,46]. Patients with significant central sensitization may not experience the expected pain relief from surgery, and understanding this can prevent unnecessary procedures and guide more appropriate treatment options, such as physical therapy or pain management strategies that focus on central mechanisms. On the other hand, some patients with nociplastic-type pain may nonetheless derive clinical benefit form joint replacement, presumably due to neutralizing an ongoing peripheral generator of pain that appears to have driven the central sensitization [47]. Spinal surgery outcomes have also been shown to be affected by the presence of nociplastic pain [48]. Thus, recognizing nociplastic pain is imperative for surgeons undertaking any kind of musculoskeletal interventions. 

#### 5.2.2. Postoperative Pain Management

Orthopedic surgeons must also be aware of the potential for nociplastic pain to complicate postoperative recovery. Patients with preexisting nociplastic pain conditions, such as fibromyalgia, may require tailored pain management strategies to prevent the development of chronic postoperative pain [46,49]. This might include the use of centrally acting medications, or non-pharmacological interventions such as mindfulness or relaxation techniques.

### 5.3. Neurology

#### 5.3.1. Differentiating Pain Syndromes

Neurologists frequently encounter patients with complex pain syndromes that do not fit neatly into traditional categories. Recognizing nociplastic pain is essential for differentiating it from neuropathic pain and other neurological disorders. For instance, in conditions like chronic headaches, migraines, or unexplained sensory disturbances, understanding the role of central sensitization can lead to more accurate diagnoses and avoid the overuse of invasive diagnostic procedures or ineffective treatments.

#### 5.3.2. Addressing Central Sensitization

Neurologists are uniquely positioned to address the central mechanisms of nociplastic pain. They can employ therapies that target the CNS, such as tricyclic antidepressants, serotonin–norepinephrine reuptake inhibitors (SNRIs), and anticonvulsants like gabapentinoids, which have shown efficacy in reducing central sensitization. Additionally, neuromodulation techniques, such as transcranial magnetic stimulation (TMS) or spinal cord stimulation, may be explored as part of a comprehensive treatment plan [50,51]. Moreover, the introduction of Calcitonin gene-related peptide (CGRP)-targeting drugs has opened a novel avenue in the treatment of chronic migraines, and may have broader implications in the management of chronic pain [52]. 

### 5.4. Psychiatry

#### 5.4.1. Management of Comorbid Mental Health Conditions

Nociplastic pain is frequently accompanied by comorbid psychiatric conditions such as depression, anxiety, and PTSD. Psychiatrists play a critical role in managing these conditions, which can exacerbate pain perception and hinder treatment progress. Recognizing nociplastic pain allows psychiatrists to address the complex interplay between pain and mental health, using interventions like CBT, pain reprocessing therapy (PRT), and acceptance and commitment therapy (ACT) to improve both psychological and physical outcomes [53,54].

#### 5.4.2. Psychological Interventions

Psychological therapies are particularly effective in managing the cognitive and emotional dimensions of nociplastic pain. For example, CBT can help patients reframe their pain experiences and reduce catastrophizing, a common issue in chronic pain sufferers [55]. PRT, which focuses on retraining the brain to reinterpret pain signals, has shown promise in reducing the severity of nociplastic pain [56]. These interventions, when combined with medical management, can provide a more comprehensive approach to treatment.

### 5.5. Rheumatology

#### 5.5.1. Comprehensive Pain Management

Rheumatologists, who are already familiar with conditions like fibromyalgia, are at the forefront of managing nociplastic pain. By recognizing nociplastic pain as a broader concept that extends beyond fibromyalgia, rheumatologists can enhance the management of other rheumatologic conditions, such as rheumatoid arthritis or lupus, where nociplastic pain may be a significant component. This understanding can lead to the more effective use of centrally acting medications and non-pharmacological therapies [57,58].

#### 5.5.2. Guideline Development and Leadership

Given their expertise in managing complex pain conditions, rheumatologists are well-positioned to lead the development of guidelines and protocols for the diagnosis and management of nociplastic pain. By advocating for the inclusion of nociplastic pain assessments in routine clinical practice, rheumatologists can help set standards that other specialties may follow, ultimately improving patient outcomes across the board.

## 6. Implications for Clinical Practice

The recognition and understanding of nociplastic pain have significant implications for clinical practice across various medical fields. Integrating the knowledge of central sensitization into the care of patients with chronic pain can transform the way healthcare providers diagnose, treat, and manage these conditions. This section outlines the key implications for clinical practice, focusing on diagnostic challenges, treatment approaches, and the potential for emerging therapies, including neurofeedback and hyperbaric oxygen therapy.

### 6.1. Diagnostic Challenges

#### 6.1.1. Subjective Nature of Nociplastic Pain

One of the primary challenges in diagnosing nociplastic pain is its subjective nature. Unlike nociceptive pain, which is often linked to observable tissue damage or inflammation, and neuropathic pain, which can be traced to objectively documented nerve damage, nociplastic pain lacks a clear peripheral pathology. This absence of visible markers can make it difficult for clinicians to identify and validate the patient’s pain experience, leading to potential underdiagnosis or misdiagnosis.

While diagnosing nociplastic pain can be challenging, due to its subjective nature and the lack of a visible peripheral pathology, the development of clinical criteria by Kosek et al. in 2022, endorsed by the IASP, has provided much-needed clarity. These criteria offer a structured approach to diagnosing nociplastic pain based on the absence of nociceptive or neuropathic origins and the presence of central sensitization features such as widespread pain, hypersensitivity to sensory stimuli, and associated fatigue and cognitive symptoms [11].

In line with these criteria, the CANPPHE (Cancer Nociplastic Pain Phenotyping) recommendations have introduced specific tools for identifying nociplastic pain in cancer survivors, emphasizing the need to distinguish nociplastic pain from pain caused by tissue damage or treatment-related neuropathy [43]. Similarly, the BACPAP (Back Pain Central Sensitization Phenotyping) guidelines have offered valuable insights into phenotyping nociplastic pain in patients with chronic low back pain, advocating for the use of comprehensive assessment tools that incorporate both physical and psychological dimensions of pain [44]. These recommendations provide practical guidance for clinicians to integrate into their diagnostic processes, helping ensure the early identification and appropriate management of nociplastic pain.

Given the complex and multifactorial nature of nociplastic pain, these multidimensional diagnostic tools should be incorporated into routine clinical practice, helping to capture not only the physical but also the emotional and cognitive components of the patient’s pain experience. Tools such as body maps can be valuable for assessing the widespread nature of pain [59], while questionnaires that evaluate comorbid symptoms can help identify the central sensitization component. Pressure pain threshold (PPT) measurement using algometry is a widely utilized method for assessing central sensitization, as it provides objective data on pain thresholds and can detect allodynia, a hallmark feature of central sensitization. Several studies have demonstrated its utility in both clinical practice and research for evaluating patients with conditions like fibromyalgia and chronic low back pain [60,61].

These tools can help clinicians differentiate nociplastic pain from other types of chronic pain, guiding treatment decisions and improving patient outcomes.

#### 6.1.2. Multidimensional Diagnostic Tools

Given the complexity of nociplastic pain, there is a growing need for multidimensional diagnostic tools [62,63] that can assess both physical and psychological aspects of the condition. These tools should be designed to capture the full spectrum of symptoms associated with nociplastic pain, including emotional and cognitive factors that contribute to the patient’s overall experience of pain. Incorporating such tools into routine practice can improve diagnostic accuracy and help tailor treatment strategies to individual patients.

### 6.2. Treatment Approaches

#### 6.2.1. Non-Pharmacological Interventions

The cornerstone of managing nociplastic pain lies in non-pharmacological interventions, which address the central sensitization at the heart of the condition. These interventions include:

− Physical therapy—Gradual, guided physical activity is crucial for patients with nociplastic pain. Exercise programs tailored to the patient’s capabilities can help reduce pain sensitivity, improve function, and prevent the deconditioning that often accompanies chronic pain [64,65];
− As mentioned above, cognitive-behavioral therapy (CBT)—CBT is highly effective in helping patients manage the psychological aspects of nociplastic pain, such as pain-related fear, anxiety, and catastrophizing. By reframing negative thought patterns, CBT can reduce the perceived intensity of pain and improve coping strategies [66,67];
− Sleep hygiene and management—Sleep disturbances are a common issue among patients with nociplastic pain, often exacerbating pain perception and worsening overall health outcomes. While sleep hygiene—such as maintaining a consistent sleep schedule, reducing caffeine intake, and optimizing the sleep environment—can be helpful, it is generally insufficient as a standalone treatment for the chronic sleep problems experienced by these patients. Instead, cognitive-behavioral therapy for insomnia (CBTi) has emerged as the gold standard for addressing sleep problems in individuals with nociplastic pain [68].

CBTi is a structured, multi-component therapy that focuses on changing the behaviors and thoughts that contribute to insomnia. It typically includes several core modules, such as sleep restriction, stimulus control, cognitive restructuring, and relaxation techniques, alongside the more basic principles of sleep hygiene [69]. This comprehensive approach has been proven to be more effective in improving sleep quality and reducing pain sensitivity than sleep hygiene alone, particularly in patients with chronic pain conditions like fibromyalgia [70].

Research indicates that improving sleep through CBTi can lead to significant reductions in pain severity, fatigue, and cognitive difficulties, which are hallmark symptoms of nociplastic pain, as well as to changes in deleterious patterns of brain connectivity [71]. Additionally, CBTi helps break the vicious cycle between poor sleep and heightened pain sensitivity by addressing maladaptive sleep-related thoughts and behaviors, thus offering a more targeted intervention than generalized sleep hygiene practices.

Incorporating CBTi into the management of nociplastic pain can have a profound impact on patients’ overall well-being, improving both their sleep and pain outcomes. Given its strong evidence base and enduring benefits, CBTi should be a primary therapeutic option for sleep problems in patients with nociplastic pain, integrated within multidisciplinary pain management strategies.

#### 6.2.2. Pharmacological Treatments

The pharmacological treatment of nociplastic pain remains a challenge, as many of the conventional analgesics used for nociceptive and neuropathic pain, such as non-steroidal anti-inflammatory drugs (NSAIDs) and opioids, have limited efficacy and may even worsen symptoms in some cases. Instead, centrally acting medications, such as serotonin–norepinephrine reuptake inhibitors (SNRIs) [72,73,74] and gabapentinoids, have been more commonly recommended for managing nociplastic pain. However, as highlighted by the recent BACPAP international expert consensus (2024), evidence supporting the effectiveness of pharmacological treatments for nociplastic pain is limited, and these treatments should be used judiciously [44].

According to the BACPAP consensus, first-line pharmacological treatments should prioritize non-opioid options with modest efficacy in reducing central sensitization. SNRIs, such as duloxetine and milnacipran, and tricyclic antidepressants (TCAs) like amitriptyline, are recommended based on their ability to modulate central pain pathways, though their effectiveness can vary among patients [75]. Gabapentinoids, including gabapentin and pregabalin, may be considered for patients with a prominent neuropathic component, but their utility in purely nociplastic pain remains less clear, with inconsistent results across clinical trials [76].

Importantly, the BACPAP guidelines emphasize that pharmacological interventions should be considered part of a comprehensive, multidisciplinary approach to nociplastic pain, rather than stand-alone solutions. Non-pharmacological therapies, such as exercise and cognitive-behavioral therapy (CBT), are often equally or more effective in improving long-term outcomes.

#### 6.2.3. Avoidance of Ineffective Treatments

Traditional analgesics like non-steroidal anti-inflammatory drugs (NSAIDs) and opioids are generally less effective in treating nociplastic pain, and may even exacerbate symptoms in some cases. Understanding the central nature of nociplastic pain can prevent the overuse of these medications, reducing the risk of side effects and dependency. Instead, treatment should focus on therapies that specifically target central sensitization.

### 6.3. Emerging Therapies

The field of emerging therapies for nociplastic pain is rapidly evolving, but many of these interventions are still in the early stages of clinical validation. While some novel treatments show promise, it is important to adopt a balanced and cautious view of their potential benefits, as highlighted in the above-mentioned BACPAP expert consensus recommendations.

Neurofeedback and Neuromodulation Techniques: Neurofeedback [44] and techniques like transcranial magnetic stimulation (TMS) [77] and spinal cord stimulation have garnered attention for their potential to modulate central nervous system activity and reduce central sensitization in nociplastic pain patients. However, the BACPAP consensus points out that while early results are promising, more rigorous and large-scale clinical trials are needed to determine the long-term efficacy and safety of these therapies. These interventions may be suitable for selecting patients who have not responded to conventional treatments, but they should not be considered first-line therapies at this stage.

Hyperbaric Oxygen Therapy (HBOT): Some studies suggest that HBOT may reduce central sensitization and improve symptoms in fibromyalgia patients [78,79], but the overall evidence remains weak, and the treatment is not widely endorsed by international guidelines at present. The BACPAP consortium recommends that HBOT be considered experimental until more conclusive data are available, and its use should be limited to controlled clinical trial settings. While therapies such as Hyperbaric Oxygen Therapy (HBOT) and neuromodulation techniques (e.g., transcranial magnetic stimulation) show promise in modulating central sensitization and reducing nociplastic pain, there is a potential risk in reinforcing the patient’s belief that their pain has a purely peripheral origin. It is important that these therapies be integrated into a broader biopsychosocial approach to pain management, ensuring that patients understand the central nervous system’s role in nociplastic pain and do not become overly reliant on physical interventions.

Cannabinoids and Psychedelics: Cannabinoids and psychedelics have emerged as potential treatments for chronic pain, including nociplastic pain, due to their effects on central nervous system modulation. However, as noted by the BACPAP consensus, robust evidence supporting their routine use is currently lacking. While small studies suggest these compounds may provide pain relief for certain patients, their safety profiles, particularly regarding long-term use, are not yet fully understood [80,81]. Thus, they should be approached cautiously, and patients should be informed of the experimental nature of these treatments.

In light of these emerging therapies, it is important for clinicians to manage patient expectations and to continue advocating for multidisciplinary approaches that incorporate both pharmacological and non-pharmacological strategies. Future research will help clarify the role of these therapies in managing nociplastic pain, but for now, they should be regarded as supplementary to established treatments rather than primary options.

#### Personalized Medicine Approaches

Advances in genomics and neuroimaging may eventually lead to more personalized approaches to treating nociplastic pain. By identifying specific biomarkers or genetic predispositions, clinicians could tailor treatments to individual patients, improving outcomes and reducing the trial-and-error approach currently common in pain management.

## 7. Future Directions

As the understanding of nociplastic pain continues to evolve, there are several key areas that warrant further exploration and development. These future directions focus on advancing research, improving clinical practices, and fostering interdisciplinary collaboration to enhance the diagnosis, treatment, and overall management of nociplastic pain.

### 7.1. Research Needs

#### 7.1.1. Biomarkers and Diagnostic Tools

One of the most pressing needs in the field of nociplastic pain is the development of reliable biomarkers and diagnostic tools. Current diagnostic criteria rely heavily on clinical assessment, which can be subjective and prone to variability. The identification of specific biomarkers—whether genetic, biochemical, or neuroimaging-based—would provide a more objective means of diagnosing nociplastic pain, and could lead to the earlier and more accurate identification of affected individuals [82]. Research into neuroimaging techniques, such as functional MRI (fMRI) and positron emission tomography (PET), as well as advancements in genomics, could play a crucial role in this endeavor.

#### 7.1.2. Leveraging Large Databases

The use of large-scale databases, such as the UK Biobank [83] and the Human Phenotype Project [84], holds significant potential in advancing our understanding of nociplastic pain. These extensive datasets provide a wealth of genetic, environmental, and phenotypic information that can be analyzed to identify potential risk factors, biomarkers, and pathways involved in nociplastic pain. By mining these databases, researchers can uncover patterns and associations that may not be apparent in smaller studies, leading to more targeted and personalized approaches to treatment. Additionally, these resources can help in understanding the heritability of and genetic predisposition to nociplastic pain, which could inform both prevention and intervention strategies.

#### 7.1.3. Mechanistic Studies

Further research is needed to elucidate the precise mechanisms underlying nociplastic pain. While central sensitization is a well-established concept, the specific pathways and processes that contribute to this heightened pain sensitivity are not fully understood. Studies focusing on the role of the immune system, neuroinflammation [85,86], and neuroplasticity could provide deeper insights into the pathophysiology of nociplastic pain. Additionally, exploring the interactions between central and peripheral mechanisms, particularly in conditions like small fiber neuropathy and autonomic dysfunction, could clarify the contributions of these factors to the overall pain experience.

#### 7.1.4. Clinical Trials for Emerging Therapies

As novel therapies such as neurofeedback, hyperbaric oxygen therapy, cannabinoids, and psychedelics gain interest, rigorous clinical trials are essential to determine their efficacy and safety in treating nociplastic pain. These studies should focus not only on pain relief, but also on the broader impact on patients’ quality of life, functional abilities, and psychological well-being. Moreover, understanding which subgroups of patients are most likely to benefit from these therapies will be crucial for developing personalized treatment approaches.

### 7.2. Clinical Practice Improvements

#### 7.2.1. Integrating Multidisciplinary Care

The management of nociplastic pain requires a multidisciplinary approach that involves collaboration among various specialties, including rheumatology, neurology, psychiatry, pain management, and primary care. Future clinical practice should emphasize the integration of care teams to address the multifaceted nature of nociplastic pain. This approach would ensure that patients receive comprehensive care that addresses not only their physical symptoms, but also their emotional, cognitive, and social needs.

#### 7.2.2. Education and Training

There is a growing need for education and training programs that equip healthcare professionals with the knowledge and skills to recognize and manage nociplastic pain. These programs should be incorporated into medical curricula and continuing education for all relevant disciplines. By enhancing the awareness and understanding of nociplastic pain, clinicians can improve diagnostic accuracy and provide more effective, patient-centered care. Training should also focus on the appropriate use of both pharmacological and non-pharmacological treatments, as well as the importance of interdisciplinary collaboration.

#### 7.2.3. Patient Education and Self-Management

Empowering patients with knowledge about nociplastic pain and self-management strategies is critical for improving outcomes. Future clinical practice should place a stronger emphasis on patient education, helping individuals understand their condition and the various treatment options available to them. Self-management programs that include components like exercise, mindfulness, cognitive-behavioral strategies, and pain education can enhance patients’ ability to cope with their pain and improve their overall quality of life.

### 7.3. Interdisciplinary Collaboration and Policy Advocacy

#### 7.3.1. Fostering Collaboration

The complexity of nociplastic pain demands a collaborative effort among researchers, clinicians, and policymakers. Future initiatives should focus on fostering interdisciplinary research that brings together expertise from different fields to tackle the challenges of nociplastic pain. Collaborative research networks and consortia could facilitate the sharing of data, resources, and knowledge, accelerating the pace of discovery and innovation.

#### 7.3.2. Policy Advocacy

Advocacy for policy changes that recognize the burden of nociplastic pain and support research and clinical care is essential. Policymakers should be encouraged to fund research into nociplastic pain, support the development of new diagnostic and treatment tools, and ensure that healthcare systems are equipped to provide comprehensive care for patients with chronic pain. Advocacy efforts should also focus on reducing the stigma associated with chronic pain conditions, promoting a more compassionate and understanding approach to patient care.

#### 7.3.3. Global Perspectives

Nociplastic pain is a global health issue that affects individuals across diverse populations and healthcare systems. Future research and clinical practice should consider the cultural, social, and economic factors that influence the experience and management of nociplastic pain. Collaborative efforts at an international level can help develop strategies that are culturally sensitive and adaptable to different healthcare settings, ensuring that all patients have access to effective pain management.

## 8. Conclusions

The recognition and understanding of nociplastic pain represent a critical advancement in the field of chronic pain management. This concept shifts the focus from traditional models that emphasize peripheral damage to a more nuanced understanding that centers on the central nervous system’s role in pain processing. Nociplastic pain, characterized by central sensitization and altered neural connectivity, poses unique challenges for diagnosis and treatment, but it also offers new opportunities for improving patient care.

Integrating the concept of nociplastic pain into clinical practice is essential across multiple disciplines, including primary care, orthopedics, neurology, psychiatry, and rheumatology. By recognizing the central mechanisms at play, healthcare providers can adopt more effective, multidisciplinary approaches that address both the physical and psychological aspects of chronic pain. The inclusion of emerging therapies such as neurofeedback and hyperbaric oxygen therapy, along with advancements in personalized medicine, holds promise for patients who have not responded to conventional treatments.

Looking forward, the future of nociplastic pain management will be shaped by continued research into the underlying mechanisms of pain, the development of reliable biomarkers and diagnostic tools, and the exploration of novel therapeutic approaches. Large-scale databases like the UK Biobank and the Human Phenotype Project will play a pivotal role in uncovering the genetic, environmental, and phenotypic factors that contribute to nociplastic pain, paving the way for more targeted and individualized treatments.

Moreover, the importance of interdisciplinary collaboration cannot be overstated. The complexity of nociplastic pain demands that researchers, clinicians, and policymakers work together to develop and implement comprehensive strategies that improve patient outcomes. Advocacy efforts are also crucial to ensure that the healthcare system recognizes the burden of nociplastic pain and provides the necessary resources to address it. Ultimately, the goal is to enhance the quality of life for individuals living with nociplastic pain. By advancing our understanding of this condition and refining our approaches to its management, we can offer more compassionate, effective, and patient-centered care. This is not just an academic or clinical challenge; it is a call to action for all who are involved in the care of patients with chronic pain.
